# Impact of the ‘reserved therapeutic space’ nursing intervention on patient health outcomes: An intervention study in acute mental health units

**DOI:** 10.1002/nop2.1750

**Published:** 2023-04-21

**Authors:** Antonio R. Moreno‐Poyato, Khadija El Abidi, Teresa Lluch‐Canut, Montserrat Cañabate‐Ros, Montserrat Puig‐Llobet, Juan F. Roldán‐Merino

**Affiliations:** ^1^ Department of Public Health, Mental Health and Maternal and Child Health Nursing, Nursing School Universitat de Barcelona L'Hospitalet de Llobregat Spain; ^2^ Institut de Neuropsiquiatria i Addiccions Hospital del Mar Barcelona Spain; ^3^ TXP Research Group, Universidad Cardenal Herrera‐CEU CEU Universities Castelló de la Plana Spain; ^4^ Hospital Clínico Universitario Valencia Valencia Spain; ^5^ Campus Docent Sant Joan de Déu Fundació Privada, School of Nursing University of Barcelona Barcelona Spain

**Keywords:** mental health, nurse–patient relationship, nursing intervention, psychiatric nursing

## Abstract

**Aims:**

To evaluate the effectiveness of the ‘reserved therapeutic space’ intervention for improving the nurse–patient therapeutic relationship in acute mental health units in Spain.

**Design:**

Multicentre intervention study with control group.

**Methods:**

The study will be carried out in 12 mental health units. The ‘reserved therapeutic space’ intervention to be tested has been co‐designed and validated by both nurses and patients. The quality of the therapeutic relationship, the care received and perceived coercion among patients will be assessed. An estimated 131 patients per group are expected to participate. Funding was granted by the Instituto de Salud Carlos III. Co‐financed by the European Union (European Regional Development Fund (ERDF) (PI21/00605)) and College of Nurses of Barcelona (PR‐487/2021). The proposal was approved by all the Research Ethics Committees of participating centres.

**Results:**

This project will lead to changes in clinical practice, transforming the current models of organization and care management in mental health hospitalization units. No patient or public contribution.

## 1 INTRODUCTION

The therapeutic relationship is internationally considered to be at the core of mental health nursing care (Moreno‐Poyato et al., 2016). Indeed, the quality of its establishment is associated with better evidence‐based practice on behalf of nurses (Moreno‐Poyato, Casanova‐Garrigos, et al., 2021). Moreover, the therapeutic relationship is used as a vehicle for improving the health of people with mental health problems (Zugai et al., 2015). However, the correct development and maintenance of the therapeutic relationship is complex and even more so in the context of inpatient mental health units (Molin et al., 2018; Moreno‐Poyato, El Abidi, Rodríguez‐Nogueira, et al., 2021). Therefore, it is necessary to design and evaluate interventions aimed at improving the therapeutic relationship in mental health units through the participation of all the main actors, both patients and professionals, and based on solid theoretical foundations and methodologically consistent designs (Hartley et al., 2020).

## 2 BACKGROUND

In recent years, the national strategies on mental health in Catalonia and Spain have been aimed at person‐centred care and personal recovery. Thus, one of the main strategic lines of action is to improve care practices in relation to people's rights and to strengthen new capacities for the participation and empowerment of service users (Generalitat de Catalunya, Departament de Salut, 2017; Spanish Ministry of Health, 2022). In Spain, from a training point of view, one of the main challenges is to adapt the supply of training places for specialist nurses, as mental health nursing is recognized with an official postgraduate specialized training qualification (Spanish Ministry of Health, 2022). Along the same lines, the policy guidelines are committed to improving practice by promoting the active participation of professionals and users through the principles of dialogue, active listening and the person with mental health problems as a subject of rights (Generalitat de Catalunya, Departament de Salut, 2017; Spanish Ministry of Health, 2022). There is no doubt that it is through the therapeutic relationship that nurses in mental health care settings can enhance person‐centred practice (Tolosa‐Merlos et al., 2022). It is, therefore, a challenge for nurses to respond to these needs in the context of Spanish mental health inpatient units, where some users are admitted involuntarily (Wasserman et al., 2020) and remain in the hospital for an average stay of 15–16 days (Spanish Ministry of Health, 2022).

Multiple factors affect the quality of the nurse–patient therapeutic relationship in mental health units. First, factors related to the practice environment, such as the existence of biomedical models in the units (Roviralta‐Vilella et al., 2019), make it difficult to implement psychosocial interventions that improve the therapeutic relationship (Raphael et al., 2021). In this sense, other important environmental factors that have traditionally been identified as barriers to establishing quality relationships in the units are the lack of time on behalf of the nurses, either due to administrative tasks or due to a low nurse–patient ratio (Kingston & Greenwood, 2020). In addition, nurses consider the lack of leadership and support from supervisors to be an important limiting factor (Moreno‐Poyato, Rodríguez‐Nogueira, et al., 2021; Moreno‐Poyato, Roviralta‐Vilella, et al., 2021). Moreover, it should be considered that many units maintain outdated regulations and have structures that do not favour spaces for the relationship with the public (Adler, 2020; Tolosa‐Merlos et al., 2021). Most mental health units are locked units, which nurses perceive was causing their therapeutic relationship with the patient to get lost (Missouridou, Resoulai, et al., 2021). A good therapeutic relationship depends on the trust given to patients by keeping the doors open (Missouridou et al., 2022; Missouridou, Xiarhou, et al., 2021). Existing evidence also shows that nurses in acute mental health wards lack knowledge about how to engage effectively and in a way that helps their patients. Nurses affirm that this affects their education and training in the therapeutic relationship (McCluskey et al., 2022). Some studies have highlighted the importance of developing therapeutic relationship competence during training. Thus, there is a need to develop mental health professionals' therapeutic relationship knowledge and skills in professional training in acute mental health care (Liao & Murphy, 2019; Ruud & Friis, 2022). Consequently, all these factors contribute to patients reporting that nurses are often inaccessible and unavailable, indicating a lack of communication and a lack of opportunity to participate in their care (Rio et al., 2020). These aspects are basic for an effective nurse–patient therapeutic relationship (Aznar‐Huerta et al., 2021).

In recent years, different strategies have been implemented to improve the nurse–patient therapeutic relationship in mental health hospitalization units (McAllister et al., 2021; Molin et al., 2018; Moreno‐Poyato et al., 2018; Moreno‐Poyato, Rodríguez‐Nogueira, et al., 2021; Moreno‐Poyato, Roviralta‐Vilella, et al., 2021). Spaces for joint activities have been designed and implemented in the unit in a group setting to facilitate therapeutic engagement between nurses and patients (Molin et al., 2018). Combined strategies such as reflective staff groups and the use of individual daily interactions with patients have been used (Moreno‐Poyato et al., 2019). Group interventions in the form of clinical sessions for staff on patient case discussions have been carried out to improve understanding of the factors influencing patient behaviour (Berry et al., 2016). However, there is insufficient evidence that any intervention has demonstrated acceptable effectiveness (Hartley et al., 2020). Hence, the recommendations are aimed at constructing and evaluating interventions that involve the participation of both users and professionals, that are based on solid theoretical foundations and that propose methodologically consistent designs (Hartley et al., 2020).

Consequently, as a result of a participatory project with nurses from two mental health units, a nursing intervention was designed, implemented and evaluated, to enhance individualized encounters with their patients, obtaining excellent quantitative and qualitative results in the improvement of the therapeutic relationship (Moreno‐Poyato et al., 2018, 2019). Subsequently, 198 nurses from 18 mental health units participated in a new study in which they agreed on, implemented and evaluated a very similar intervention, which they called ‘reserved therapeutic space’ (RTS), emerging as part of one of the strategies to improve the therapeutic relationship of nurses with their patients (Moreno‐Poyato, Rodríguez‐Nogueira, et al., 2021; Moreno‐Poyato, Roviralta‐Vilella, et al., 2021). However, given the recommendations to design and validate the intervention with those people who will later form a relevant part of the intervention, a new study was carried out. This new study aimed at exploring the feasibility and validity of the intervention with participants with first‐person experience, belonging to two social activist organizations in the field of mental health, thus incorporating the patients' perspective of the intervention as protagonists (Moreno‐Poyato, El Abidi, Rodríguez‐Nogueira, et al., 2021).

## 3 THE STUDY

### 3.1 Aims

The main objective of this study is to evaluate the effectiveness of the RTS intervention in terms of its impact on the improvement of the nurse–patient therapeutic relationship in mental health inpatient units.

The secondary objectives of this study are to determine the effectiveness of the RTS in terms of its impact on:

- The quality of the therapeutic relationship of nurses with their patients as perceived by people hospitalized in acute mental health units.
- The level of quality of psychiatric care perceived by people hospitalized in acute mental health units.
- The degree of coercion perceived by persons hospitalized in acute mental health units.

### 3.2 Hypotheses

The RTS intervention improves the therapeutic relationship between nurses and patients hospitalized in mental health units.

Hypothesis 1 Inpatients receiving the RTS intervention by their referent nurse will have a higher difference in mean score relative to inpatients receiving standard care on the therapeutic relationship measure using the Working Alliance Inventory‐Short scale (WAI‐S).

Hypothesis 2 Patients receiving the RTS intervention by their referring nurses will obtain a higher mean score difference compared to patients receiving standard care when measuring psychiatric quality of care using the Quality in Psychiatric Care (QPC) scale.

Hypothesis 3 Patients receiving the RTS intervention by their referring nurses will obtain a lower mean score difference compared to patients receiving standard care on the measurement of perceived coercion using the Coercion Experience Scale (CES) instrument.

### 3.3 Design

**FIGURE 1:**
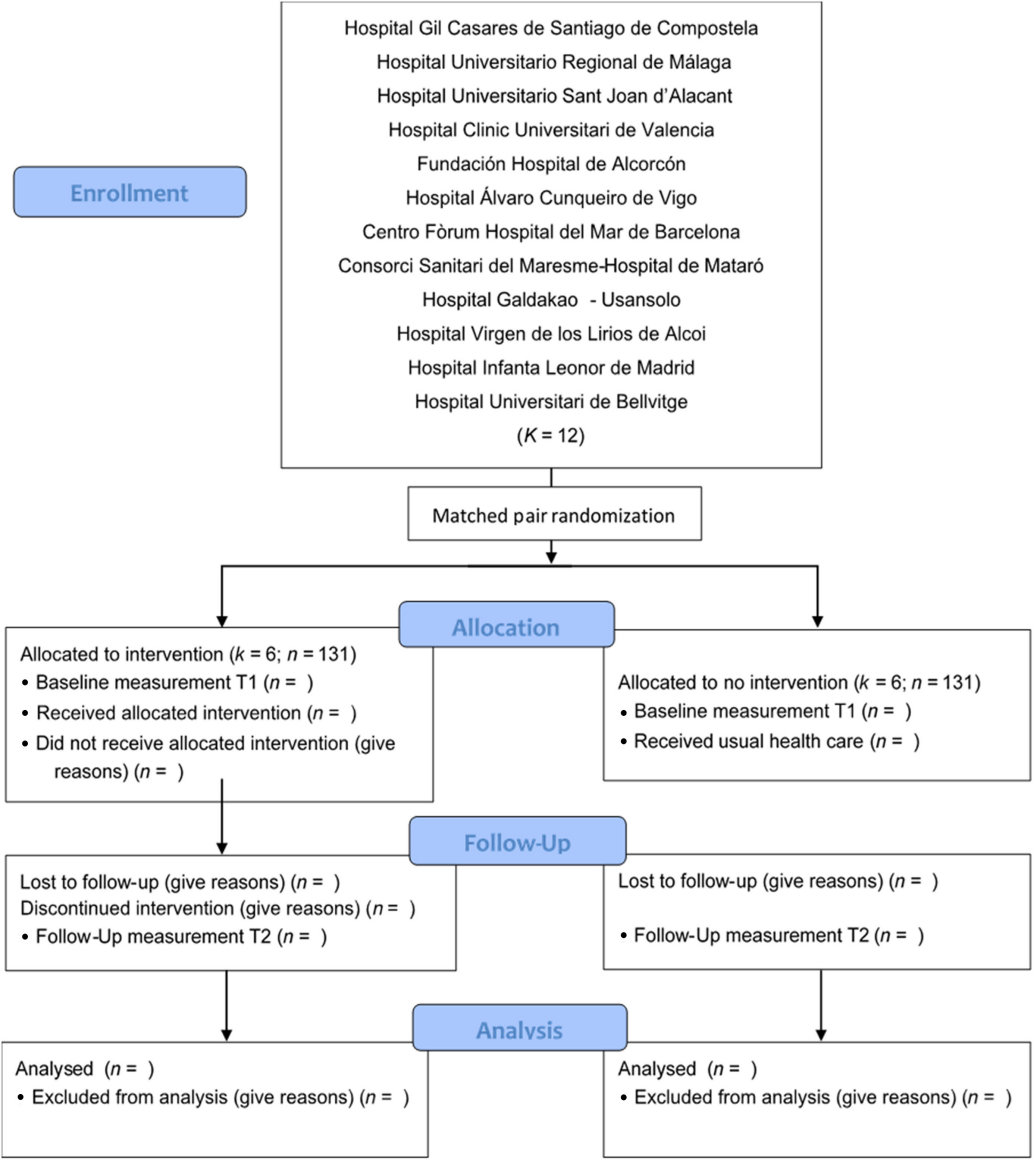
CONSORT Flow diagram RTS_MHNursing_Spain.

A multicentre intervention study with a control group aimed at evaluating the effectiveness of the RTS intervention under real clinical practice conditions versus usual care for patients in acute mental health units in Spain (Figure 1).

### 3.4 Study setting and population

The study population will be patients hospitalized in acute mental health units in Spain. A total of 12 mental health hospitalization units will participate. The mental health units have been invited through the Spanish Association of Mental Health Nursing following territorial criteria of representativeness by autonomous community.

### 3.5 Inclusion and exclusion criteria

Eligible subjects will be adults hospitalized in mental health inpatient units who voluntarily consent to participate in the study. Participants will be excluded if they present a language barrier, mechanical restraint, contraindication by the clinical referent, cognitive impairment or intellectual disability at the time of recruitment. To maximize the external validity of the study, no other exclusions will be made.

### 3.6 Sample size

Accepting an alpha risk of 0.05 and a beta risk of 0.2 in a bilateral contrast, 131 subjects in the first group and 131 in the second group are required to detect a difference equal to or greater than 3 units. The common standard deviation is assumed to be 10 with a correlation coefficient between the initial and final measurement of 0.7. A loss‐to‐follow‐up rate of 20% was estimated.

### 3.7 Randomization

Given the difficulties due to the evaluation conditions in real clinical practice for a traditional randomization of the subjects or a randomization by clusters, and bearing in mind that not all the nurses in the units will participate in the intervention, the units will be paired by similarity of structural characteristics in terms of number of beds and will be randomized through a computer program to an intervention or control group. Randomization will take place prior to the training of the nurses and the recruitment of the subjects. The intervention will be blinded at the subject level.

### 3.8 The intervention

The RTS (Moreno‐Poyato, El Abidi, Lluch‐Canut, et al., 2021) is an intervention that will be carried out by nurses from mental health hospitalization units together with the hospitalized persons for whom they are referents. To ensure the fidelity of the intervention, the nurses of the centres selected for the intervention, and who agree to collaborate in the study, will receive complementary training from the research team prior to initiating recruitment, in addition to a brief guide to the implementation of the intervention. Likewise, the intervention will be interrupted if the assigned nurse were to take sick leave or transfer to another service and/or if the patient were to resign to continue the study. Furthermore, to improve compliance with the intervention protocol, in addition to other recommendations, the guidelines include recommendations for nurses to record the sessions in the patient's clinical history.

The main objective of the RTS intervention is to enhance the therapeutic bond of trust, to enable the agreement of objectives and interventions in a shared manner in relation to their recovery process in the unit. The intervention is carried out through individual meetings between the nurse and the patient in a comfortable and intimate space, where there are no interruptions and which is also chosen by the person who is hospitalized. The content of the spaces must be constructed individually according to the patients' concerns at each moment of the process.

The intervention consists of three phases:

- Phase one or orientation meeting: this should take place within the first 24–72 h of hospitalization at the request of the referring nurse for the patient's stay. It is aimed at making contact and building a bond of trust. In this first meeting, the patient's main concerns should be identified and, as far as possible, an agreement should be established for the following follow‐up meetings. Possible topics to be discussed at this stage are the unit's regulations (permissions, visits, objects, etc.), the vulnerability of rights, the experience of involuntary admission or even feelings of guilt in relation to what is happening to the patient.
- Phase two, with follow‐up meetings: during the following days of the hospitalization, these meetings should take place at the patient's request and the number of meetings should be as many as the patient requests and the nurse is able to offer. To facilitate this, the nurse should demonstrate availability, so that each day at the beginning of the shift, the nurse should greet and invite the patient to meet up during the working day. Similarly, at the end of the shift, the nurse should say goodbye and invite the patient for the next working day. As for the content of the meetings during this phase, the topics to be discussed will once again be determined by the patient's concerns at the time regarding his or her hospitalization process. To facilitate the development of the meetings in this phase, the key topics are the side effects of the medication, the duration of the admission, the extra medication or the incidents in the unit.
- Phase three, with a farewell meeting: in the pre‐discharge stage of hospitalization, in this last meeting it is important for the nurse to positively reinforce the patient's evolution in the hospitalization process in order to empower the person, but also to help to provide guidance at the time of discharge from the unit. In this meeting, it is especially important to provide information on resources and continuity of care outside the acute care unit. Likewise, to resolve doubts and concerns about future plans and to recommend strategies for the prevention of relapses.

### 3.9 Outcome measures

- The Working Alliance Inventory Short Questionnaire (WAI‐S), patient version. This is an instrument for measuring the working alliance, and therefore, the therapeutic relationship between professional and patient (Horvath & Greenberg, 1989). The short version of the WAI has 12 items, each item is rated by the professional and the patient on a Likert scale ranging from 1 (never) to 7 (always). This instrument is made up of three factors: bonding, agreement on objectives and agreement on tasks. The Spanish adaptation of Andrade‐González and Fernández‐Liria (2015) presents Cronbach's *α* values of 0.93 for the total scale and 0.85, 0.81 and 0.90 for each of the factors.
- The Quality in Psychiatric Care scale (QPC: Schröder et al., 2007) assesses the quality of care and is the best suited to evaluate the quality of care in psychiatric units. The QPC contains 30 items and each item is evaluated by means of a Likert scale with four response options, where 1 indicates total disagreement and 4 indicates total agreement. It is composed of six dimensions: therapeutic relationship, patient participation, support received, environment, safety and hospital discharge. The maximum score is 120 points, and the minimum is 30 points. A high score in each dimension or in the total scale would indicate a very good perception of quality on behalf of the patient. The Spanish version will be used (Sanchez‐Balcells et al., 2021) which presents a Cronbach's *α* of 0.94 for the total scale and for five of the six dimensions it was over 0.7, except for the dimension measuring the environment, which was 0.67.
- The Coercion Experience Scale (CES‐18: Bergk et al., 2010). This scale assesses the subjective experience of coercion during psychiatric hospitalization. The CES‐18 in its short version consists of 18 items that are divided into 2 dimensions, one measuring coercion and humiliation and the other fear, on a five‐point Likert‐type scale (1‐never and 5‐always); where participants rate and record their own evaluation and self‐perception of the coercive intervention experienced. This instrument was validated in a Spanish population by Aguilera‐Serrano et al. (2019), which presents a Cronbach's *α* of 0.94 for the total scale and 0.93 and 0.71 for its two factors.

### 3.10 Recruitment and data collection

Once the centres have been randomized and the collaborating nurses of the units where the intervention will be carried out have been trained, the person from the research team coordinating the study in each unit will be responsible for informing the patients who meet the inclusion criteria between 24 and 72 h from the time of admission about the objectives of the study and will obtain informed consent. Patients will be recruited consecutively and will receive a confidential code assigned by the project coordinator, which will be their identification for the data collection dossiers. In the case of patients from centres chosen for the intervention, one of the nurses who has received the training will be assigned to the patient. In the case of control centres, the usual process of assigning a nurse from the unit will be followed and the nurse will not have any notification in this regard. In both cases, once the first meeting or welcome interview by the referring nurse in the unit has taken place, the baseline assessment (T1) will be carried out by a nurse who is not involved in the patient's care process and who has been previously trained in the measurement instruments. The baseline assessment will be included in the T1 dossier and will consist of administering the WAI‐S and QPQ instruments. Prior to discharge, the same person outside the care process of the hospitalized person will administer the instruments to the user for the follow‐up assessment included in the T2 dossier, which will include the WAI‐S, the QPC and the CES (T2). The remaining data will be obtained from the patient's clinical history and will be collected by the centre's principal investigator through the data collection dossier.

### 3.11 Data analysis

The research team will have a common space through a web platform to create and manage data. In this platform, a database will be generated for each centre to which only the researchers of each centre and the methodological team will have access. Also, a common database will be generated for all the centres to which only the project coordinator and the members of the methodological team will have access. The researchers of each centre will register the data in the specific database of each centre and will send the results in parallel to the study coordinator, who, through the methodological team, will also register the data in the common database. This will ensure the double entry of the data.

Descriptive statistics will be used to describe the sociodemographic and clinical characteristics of the patients through means and standard deviations (SD); frequencies and proportions. The chi‐square test (χ^2^) and the t‐test for independent samples will be used to detect any significant differences between the intervention and control groups on baseline variables. A repeated measures analysis of variance will be performed to analyse the relationship of the intervention with each of the dependent variables by introducing sociodemographic and clinical characteristics into the model.

To evaluate the effectiveness of the intervention, a multivariate analysis of covariance analysis (MANCOVA) will be performed, introducing the pretest‐posttest differences in the quality of the therapeutic relationship, the quality of psychiatric care and the perception of coercion as dependent variables. Statistical analyses will be performed using SPSS v27.

### 3.12 Ethical considerations

This project has received approval by the bioethics committee of the ‘REDACTED’ and by each of the reference ethics committees for each of the other participating institutions. The consequent authorization will be requested from all participants for the public result that will be given to the data, preserving anonymity and strict confidentiality at all times, and therefore the name of the participants will be coded and numbered. The data obtained will be incorporated into an electronic database and the information provided will be treated as completely confidential. Any data or name that might reveal the identity of the informants will be eliminated. The persons who participate will do so voluntarily, signing the corresponding consent of acceptance of the study conditions, with the right to withdraw from the study at all times.

### 3.13 Validity and reliability/rigour

#### 3.13.1 Study protocol

The methodology for conducting this protocol follows the guidelines set out in the 2013 SPIRIT Statement (Chan et al., 2013). This study has been registered in www.clinicaltrials.gov under the code NCT05220566. Funding was granted in 2021 by the Instituto de Salud Carlos III (PI21/00605)), and by the College of Nurses of Barcelona (PR‐487/2021), after a peer‐reviewed funding process. This project is also co‐funded by the European Regional Development Fund (ERDF).

#### 3.13.2 Study intervention

The intervention under study is described following the TIDieR (Template for Intervention Description and Replication) Checklist. In addition, a brief guide to the intervention has been published to facilitate its implementation in mental health units (Moreno‐Poyato, El Abidi, Lluch‐Canut, et al., 2021).

## 4 DISCUSSION

People who require acute mental health hospitalization are undergoing a period of frailty and vulnerability at all levels. In many cases, their hospitalization is involuntary, and many people feel that their rights have been and are constantly violated. In most cases, given their cognitive, emotional and/or behavioural state, they require intensive nursing care. In this regard, the nurse as a health professional who will continuously accompany the patient throughout this hospitalization process must ensure their well‐being and has a special relevance and impact in terms of improvement and early recovery both at the psychopathological level and in terms of adaptation, acceptance and empowerment of the person in relation to the mental health problem they are suffering from. To this end, mental health nurses use a fundamental tool called the therapeutic relationship. This allows them to know, understand, agree and work with the patient's health needs throughout the hospitalization process.

Consequently, this project aims to evaluate the effectiveness of a nursing intervention focused on the therapeutic nurse–patient relationship in acute mental health units in Spain and its impact on the health outcomes of hospitalized patients. The RTS intervention aims to enable nurses to delve into the more experiential aspects of the hospitalization process of patients and their needs during hospitalization. In this manner, a greater knowledge and understanding of the patient will allow nursing care to be adapted to a greater extent to their situation and, therefore, contribute to the improvement of the health outcomes and to improved quality of care for the people who undergo the hospitalization process. Thus, the intervention is fully in line with the person‐centred care model and falls within the framework of the recovery model in force in national and international mental health policies.

### 4.1 Strengths and limitations

This study has several limitations. The first limitation is the impossibility of randomizing the subjects to the groups due to pragmatic factors in the real clinical practice condition. Therefore, we decided to pair centres with similar characteristics and subsequently randomize each centre to the intervention or control group.

Another possible limitation is that the evaluator will know which group each centre belongs to, for this reason, we have decided to train these evaluators beforehand so that they collect the information in the same manner, and it is also foreseen that these evaluators will be external to the research team.

## 5 CONCLUSION

This project will derive in changes in the practice of clinical care for people with mental disorders who require hospital admission, as it fulfils the objective of gathering new scientific knowledge that will be transferred not only to clinical research, but also to care practice, promoting the quality of patient care and transforming the current models of organization and care management of mental health hospital wards, providing nurses with a valid and effective intervention to improve the therapeutic relationship, the quality of care and patients' perception of coercion. These are highly relevant aspects of current mental health policies that promote person‐centred care, based on the recovery model and shared decision making.

## FUNDING INFORMATION

Funding was granted in 2021 by the Institute of Health Carlos III (PI21/00605, Ministry of Science and Innovation) and in October 2021 by the College of Nurses of Barcelona (PR‐487/2021), after a peer‐reviewed funding process. This project is also co‐funded by the European Regional Development Fund (ERDF).

## CONFLICT OF INTEREST STATEMENT

The authors have no conflict of interest to declare.

## Data Availability

Data sharing not applicable—no new data generated.
